# Epitaxial highly ordered Sb:SnO_2_ nanowires grown by the vapor liquid solid mechanism on m-, r- and a-Al_2_O_3_†

**DOI:** 10.1039/c9na00074g

**Published:** 2019-04-09

**Authors:** M. Zervos, N. Lathiotakis, N. Kelaidis, A. Othonos, E. Tanasa, E. Vasile

**Affiliations:** Nanostructured Materials and Devices Laboratory, School of Engineering, University of Cyprus PO Box 20537 Nicosia 1678 Cyprus zervos@ucy.ac.cy; Theoretical and Physical Chemistry Institute, National Hellenic Research Foundation Vass. Constantinou 48 GR-11635 Athens Greece; Faculty of Engineering, Environment and Computing, Coventry University Priory Street Coventry CV1 5FB UK; Laboratory of Ultrafast Science, Department of Physics, University of Cyprus P.O. Box 20537 Nicosia 1678 Cyprus; Department of Science and Engineering of Oxide Materials and Nanomaterials, Politehnica University of Bucharest 313 Splaiul Independentei Bucharest 060042 Romania

## Abstract

Epitaxial, highly ordered Sb:SnO_2_ nanowires were grown by the vapor–liquid–solid mechanism on m-, r- and a-Al_2_O_3_ between 700 °C and 1000 °C using metallic Sn and Sb with a mass ratio of Sn/Sb = 0.15 ± 0.05 under a flow of Ar and O_2_ at 1 ± 0.5 mbar. We find that effective doping and ordering can only be achieved inside this narrow window of growth conditions. The Sb:SnO_2_ nanowires have the tetragonal rutile crystal structure and are inclined along two mutually perpendicular directions forming a rectangular mesh on m-Al_2_O_3_ while those on r-Al_2_O_3_ are oriented in one direction. The growth directions do not change by varying the growth temperature between 700 °C and 1000 °C but the carrier density decreased from 8 × 10^19^ cm^−3^ to 4 × 10^17^ cm^−3^ due to the re-evaporation and limited incorporation of Sb donor impurities in SnO_2_. The Sb:SnO_2_ nanowires on r-Al_2_O_3_ had an optical transmission of 80% above 800 nm and displayed very long photoluminescence lifetimes of 0.2 ms at 300 K. We show that selective area location growth of highly ordered Sb:SnO_2_ nanowires is possible by patterning the catalyst which is important for the realization of novel nanoscale devices such as nanowire solar cells.

## Abstract 2

Epitaxial, highly ordered Sb:SnO_2_ nanowires were grown by the vapor–liquid–solid mechanism on m-, r- and a-Al_2_O_3_ between 700 °C and 1000 °C using metallic Sn and Sb with a mass ratio of Sn/Sb = 0.15 ± 0.05 under a flow of Ar and O_2_ at 1 ± 0.5 mbar.

## Graphical abstract

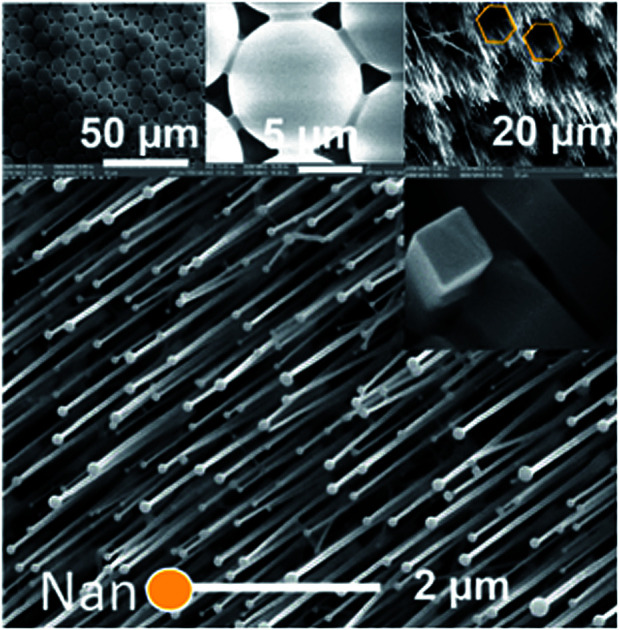

## 1 Introduction

Metal oxide semiconductor nanowires (NWs) such as Sb:SnO_2_,^1^ Sn:In_2_O_3_,^2^ Al:ZnO^3^ and In:ZnO^4^ NWs have a high conductivity but they are also capable of light emission as shown for Sn:In_2_O_3_ NWs by O' Dwyer *et al.*^5^ and Gao *et al.*^6^ Despite ongoing efforts into the growth and properties of such metal oxide (MO) NWs only a few have obtained epitaxial, ordered networks which is essential for the realization of novel nanoscale devices with improved performance like nanowire solar cells (NWSCs). More specifically Wan *et al.*^7^ has obtained ordered Sn :In_2_O_3_ NWs by homo epitaxy on an Sn:In_2_O_3_ buffer layer while Nguyen *et al.*^8^ and Gao *et al.*^6^ have obtained ordered Sn:In_2_O_3_ NWs by hetero epitaxy on m-, a- and c-Al_2_O_3_. To the best of our knowledge all other MO NWs that have been obtained previously are not oriented in an epitaxial fashion along any particular direction. It is important then to obtain earth abundant MO NWs such as Sb:SnO_2_ NWs as low cost alternatives to Sn:In_2_O_3_.

In the past we have grown SnO_2_ NWs *via* the vapor liquid solid (VLS) mechanism at 800 °C and 10^−1^ mbar which had a carrier density of the order of 10^16^ cm^−3^ and mobility of 70 cm^2^ V^−1^ s^−1^, as determined from THz conductivity spectroscopy.^9^ High conductivity SnO_2_ NWs have been obtained *via* the incorporation of Sb,^10^ Mo^11^ and F^12^ in SnO_2_ NWs, while recently, Ma *et al.*^13^ showed theoretically that a semiconductor to semimetal transition is possible *via* the incorporation of Pb in SnO_2_. However, in most cases Sb has been used as an n-type donor impurity in SnO_2_ NWs.^14–24^ All of the Sb:SnO_2_ NWs have been obtained by using metallic Sn and Sb in the past, but they were not ordered.

Nevertheless, it is necessary to point out that Mathur *et al.*^25^ has obtained un-doped SnO_2_ NWs *via* the VLS mechanism that were ordered on TiO_2_ (001) which is isostructural with the tetragonal rutile crystal structure of SnO_2_. Similarly, Kim *et al.*^26^ obtained un-doped, epitaxial, SnO_2_ NWs on TiO_2_ (101) while Leonardy *et al.*^27^ investigated the structural properties of ordered, un-doped, SnO_2_ NWs on m- and a-Al_2_O_3_ at 700 °C by using SnO under a flow of Ar at 10 mbar. Both Mathur *et al.*^25^ and Leonardy *et al.*^27^ obtained SnO_2_ NWs which were oriented in two mutually perpendicular directions while the SnO_2_ NWs of Kim *et al.*^26^ were aligned in three directions. Others like Mazeina *et al.*^28^ have grown vertical, un-doped SnO_2_ NWs *via* the VLS mechanism on c-Al_2_O_3_ at 900 °C with limited ordering and uniformity. Lateral, but un-doped SnO_2_ NWs have also been obtained by Kim *et al.*^29^ and Choi *et al.*^30^ while more recently Wang *et al.*^31^ investigated lateral SnO_2_ NWs that were aligned on the surface of m-Al_2_O_3_. All of these studies on epitaxial, ordered un-doped SnO_2_ NWs^25–30^ focused primarily on their growth and structural properties. It is imperative then to investigate the electrical and optical properties of similar Sb:SnO_2_ NWs which is critical in evaluating their potential for subsequent use in devices such as NWSCs.

Here we show that epitaxial, ordered Sb:SnO_2_ NWs can be grown *via* the VLS mechanism on m-, r- and a- oriented Al_2_O_3_ only in a narrow window of growth conditions. We describe their morphology, structural, electrical and optical properties, in detail and show that selective area location growth of ordered Sb:SnO_2_ NWs is possible which in turn is attractive for the realization of NWSCs as a low cost alternative to Sn:In_2_O_3_ NWs.

## 2 Methods

### 2.1. Epitaxial growth of Sb:SnO_2_ NWs

The Sb:SnO_2_ NWs were grown using a 1′′ hot wall, low pressure chemical vapour deposition (LPCVD) reactor, capable of reaching 1100 °C, which was fed *via* a micro flow leak valve positioned on the upstream side, just after the gas manifold which consists of four mass flow controllers. A chemically resistant, rotary pump that can reach 10^−4^ mbar was connected downstream. For the growth of the Sb:SnO_2_ NWs, metallic Sn and Sb (Aldrich, 100 Mesh, 99.9%) were weighed with an accuracy of ±1 mg. We used an excess of Sb, *i.e.* a mass ratio of Sn/Sb ≈ 0.1, and the total mass of Sb and Sn was kept fixed and equal to 100 mg or 0.1 g. Square samples of 10 mm × 10 mm c-, m-, r- and a-Al_2_O_3_ were cleaned sequentially in trichloroethylene, methanol, acetone, isopropanol, rinsed with de-ionised water, dried with nitrogen and then coated with ≈1 nm Au. The elemental Sb and Sn as well as the c-, m-, r- or a-Al_2_O_3_ substrates were loaded in the same quartz boat which was positioned at the centre of the 1′′ LPCVD reactor. The latter was pumped down to 10^−4^ mbar and purged with 1000 sccm of Ar for 10 min at 1 mbar. Subsequently the temperature was ramped up to 800 °C at 30°C min^−1^ using the same flow of Ar. Upon reaching 800 °C a flow of 10 sccm O_2_ was added to the flow of Ar in order to grow the Sb:SnO_2_ NWs over 10 min at 1 mbar, followed by cool down without O_2_. We have grown Sb:SnO_2_ NWs on c-, m-, r- and a-Al_2_O_3_ using these growth conditions and changed the growth temperature between 700 °C to 1000 °C.

### 2.2. Characterization of Sb:SnO_2_ NWs

The morphology, crystal structure and composition of the Sb:SnO_2_ NWs was determined by scanning electron microscopy (SEM), X-ray diffraction (XRD) and Energy Dispersive X-ray analysis (EDX). High resolution transmission electron microscopy (HRTEM) was carried out using a TECNAI F30 G^2^ S-TWIN operated at 300 kV. The optical properties of the Sb:SnO_2_ NWs were determined by steady state and transient absorption–transmission spectroscopy. The steady state and time resolved photoluminescence (PL) were also measured between 10 K and 300 K, while the electrical properties, *i.e.* carrier density and resistivity, were measured by the Hall effect in the Van der Pauw geometry similar to Costa *et al.*^23,24^ In particular the Sb:SnO_2_ NWs were transferred from the m-, r- or a-Al_2_O_3_ onto 10 mm × 10 mm c-Al_2_O_3_ by applying pressure. This results into a dry transfer of the ordered Sb:SnO_2_ NWs onto the c-Al_2_O_3_ and the formation of a planar interconnected network. We then deposited In contacts over the Sb:SnO_2_ NWs by thermal evaporation using a shadow mask. The Sb:SnO_2_ NWs on c-Al_2_O_3_ was not heated up during the deposition, and the In contacts had diameters of ≈1 mm at the four corners of the 10 mm × 10 mm c-Al_2_O_3_, but we did not anneal them. The Hall effect was measured using a GMW3470 Electromagnet at 0.3 Tesla. The magnetic field was calibrated with a Hirst GM08 Gaussmeter. A Keithley 2635 A current source and Keithley 2182A nano-voltmeter, controlled by Lab View were used to provide a current and measure the voltages.

## 3 Results and discussion

**Fig. 1:**
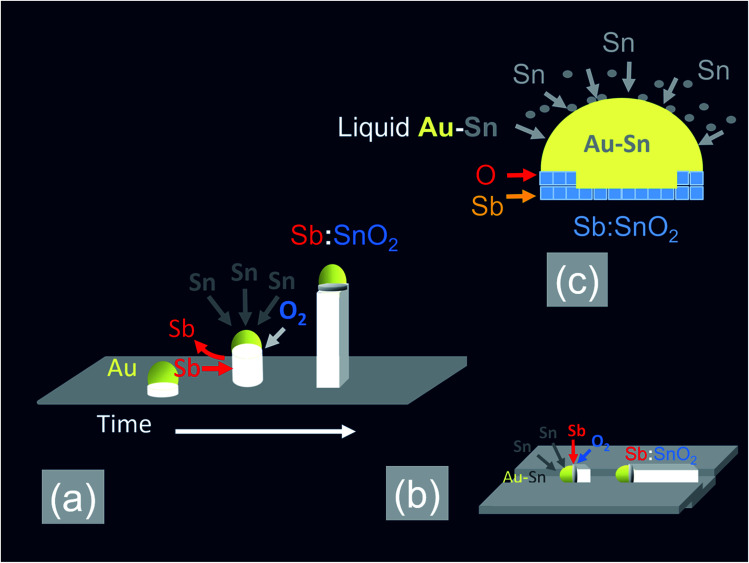
Schematic representation of the VLS growth mechanism of Sb:SnO_2_ NWs (a) bottom-up growth (b) lateral growth along a step or groove (c) Sb donor impurity incorporation mechanism.

In the past, we have shown that the reaction of Sn with O_2_ at 800 °C and 10^−1^ mbar results into a high yield and uniform distribution of SnO_2_ NWs on Si (001) or fused SiO_2_. The SnO_2_ NWs have average diameters of ≈50 nm, lengths up to ≈100 μm and grow by the VLS mechanism whereby Sn enters the Au catalyst particles on the surface of Si (001) or fused SiO_2_ and forms liquid Au:Sn particles. Upon saturation, solid SnO_2_ forms beneath the liquid Au:Sn particles *via* the reaction with O_2_ at the triple phase junction, as shown in Fig. 1(a), leading to one dimensional, bottom-up growth.^32^ However, the SnO_2_ NWs obtained on Si (001) or fused SiO_2_ were not oriented or ordered along any direction, and had a carrier density of the order of 10^16^ cm^−3^ with a mobility of 70 cm^2^ V^−1^ s^−1^, as determined from THz conductivity spectroscopy.^33^ Hence doping is required to increase their conductivity. Recently, we showed that higher carrier densities of the order of 10^18^ to 10^19^ cm^−3^ may be readily obtained in Sb doped SnO_2_ NWs grown on both Si (001) and fused SiO_2_.^34^ We obtained a high carrier density of 4 × 10^19^ cm^−3^ in the Sb:SnO_2_ NWs grown on c-Al_2_O_3_ at 800 °C and 1 mbar by adding metallic Sb to Sn but the Sb:SnO_2_ NWs were not ordered or oriented in any particular direction, similar to those obtained previously on Si (001) and fused SiO_2_.^34^ This is in contrast to the findings of Mazeina *et al.*,^28^ who obtained vertical but un-doped SnO_2_ NWs *via* the VLS mechanism on c-Al_2_O_3_ at 900 °C with a limited degree of ordering and uniformity. Similarly, we did not obtain ordered Sb:SnO_2_ NWs on c-Al_2_O_3_ by changing the growth temperature between 800 °C and 1000 °C. The epitaxial growth and ordering of Sb:SnO_2_ NWs on c-Al_2_O_3_ is not favorable in view of the fact that SnO_2_ has a tetragonal crystal structure which will not match the hexagonal crystal structure of the underlying c-Al_2_O_3_. However, we observed the formation of ordered Sb:SnO_2_ NWs on the sides of the c-Al_2_O_3_, as described in more detail in the ESI,† which is related to its specific crystallographic orientation. Therefore we carried out the growth of the Sb:SnO_2_ NWs on m- , r- and a-Al_2_O_3_ at 800 °C and 1 mbar using the same growth conditions described above.

### 3.1 Growth of Sb:SnO_2_ NWs on m-, r- and a-Al_2_O_3_

**Fig. 2:**
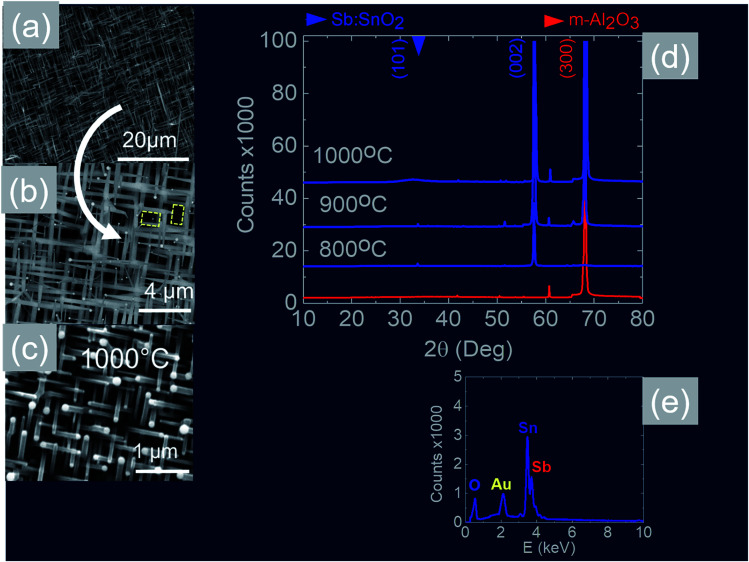
SEM images of the Sb:SnO_2_ NWs obtained at (a), (b) 800 °C and (c) 1000 °C on m-Al_2_O_3_ (d) XRD of m-Al_2_O_3_ (red) and Sb:SnO_2_ NWs (blue) grown on m-Al_2_O_3_ at 800 °C, 900 °C and 1000 °C (e) EDX of the Sb:SnO_2_ NWs.

**Fig. 3:**
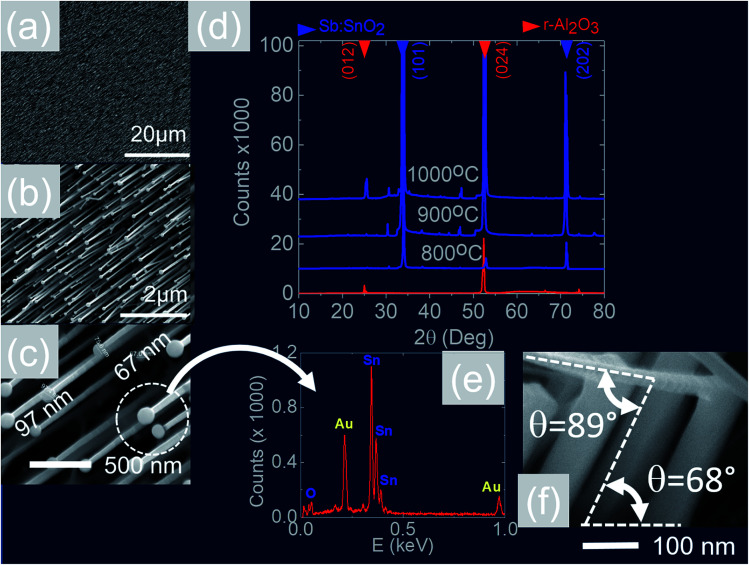
(a)–(c) SEM images of the Sb:SnO_2_ NWs obtained at 800 °C on r-Al_2_O_3_ (d) XRD of r-Al_2_O_3_ and Sb:SnO_2_ NWs grown at 800 °C , 900 °C and 1000 °C (e) EDX of an Au:Sn particle on the end of a Sb:SnO_2_ NW (f) side view showing that the first segment of the Sb:SnO_2_ NWs grows at *θ* = 68° and the second segment at *θ* = 89° with respect to the first.

**Fig. 4:**
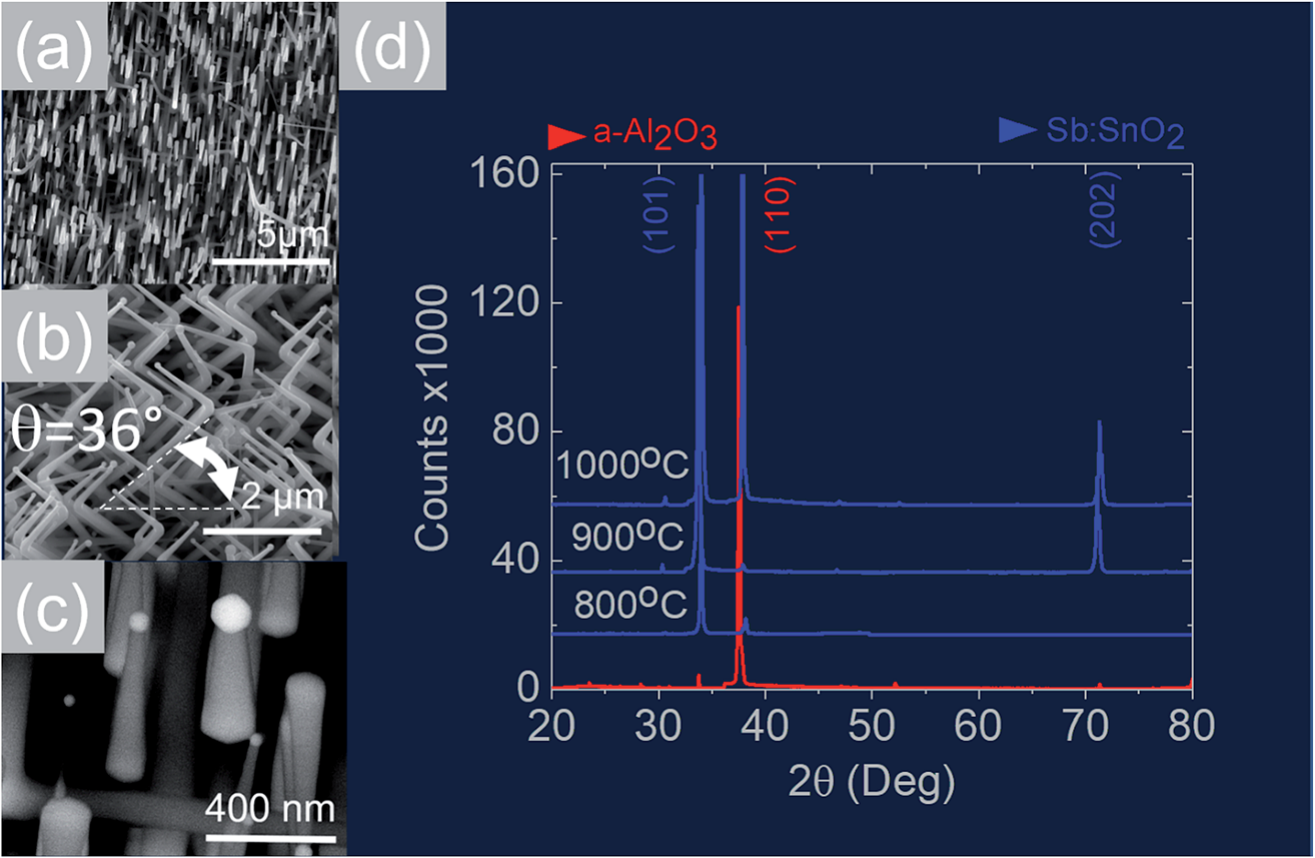
(a)–(c) SEM images of the Sb:SnO_2_ NWs obtained at 800 °C on a-Al_2_O_3_ (d) XRD of a-Al_2_O_3_ (red) and Sb:SnO_2_ NWs (blue) grown on a-Al_2_O_3_ at 800 °C, 900 °C and 1000 °C.

We readily obtained ordered Sb:SnO_2_ NWs on all three surfaces *i.e.* m-, r- and a-Al_2_O_3_, as shown in Fig. 2, 3 and 4 respectively. The Sb:SnO_2_ NWs on m-Al_2_O_3_ are aligned along two mutually perpendicular directions as shown by the SEM images in Fig. 2(a and b), but are inclined with respect to the surface, similar to the SnO_2_ NWs obtained by Mathur *et al.*^25^ and Leonardy *et al.*^27^ In contrast, the Sb:SnO_2_ NWs on r-Al_2_O_3_ are all oriented along one direction as shown in Fig. 3(a)–(c). However, upon closer inspection we find that the Sb:SnO_2_ NWs on r-Al_2_O_3_ consist of two segments. The first segment is inclined at *θ* = 68° and the second segment is at an angle of *θ* = 89°, *i.e.* nearly perpendicular to the first, as shown in Fig. 3(f). This has also been observed by Jean *et al.*^20^ who obtained Sb:SnO_2_ NWs on 10 nm Au/Si (001) *via* the VLS mechanism at 1000 °C and 1 mbar using Sn/Sb = 50, 25 and 10 under Ar and trace amount of O_2_, but their Sb:SnO_2_ NWs were not aligned in any particular direction. The formation of the second segment can be prevented by reducing the growth time as will be described in more detail later. Similarly we find that the Sb:SnO_2_ NWs on a-Al_2_O_3_ shown in Fig. 4(a–c) consist of two segments, similar to those obtained on r-Al_2_O_3_.

In all cases, we obtained a uniform distribution and ordering of Sb:SnO_2_ NWs over the 10 mm × 10 m-, r- and a-Al_2_O_3_ in reproducible way, using Sn/Sb = 0.1 at 800 °C and 1 mbar as described above. Subsequently we varied the growth temperature between 700 °C to 1000 °C in order to find if any changes occur in the growth directions and ordering. The Sb:SnO_2_ NWs obtained at 900 °C on m-Al_2_O_3_ were very similar to those obtained at 800 °C, but we find that the Sb:SnO_2_ NWs obtained at 1000 °C are short, as shown in Fig. 2(c), due to the fact that the Sn and Sb are more or less completely transferred into the gas stream under the flow of Ar during the temperature ramp, before the onset of one dimensional growth. In fact, the depletion of Sb during the temperature ramp is more significant than Sn, due to the fact that Sn has a melting point of 232 °C and vapor pressure of 10^−5^ mbar, while Sb has a higher melting point of 630 °C, but a remarkably higher vapor pressure of 10^−1^ mbar at 1000 °C. This in turn implies an important doping limitation when trying to obtain high carrier densities and conductivity in Sb:SnO_2_ NWs using metallic Sn and Sb.^34^ Likewise, the Sb:SnO_2_ NWs obtained on m-Al_2_O_3_ at 700 °C were short, similar to those obtained at 1000 °C, due to the limited supply of Sn which has a lower vapor pressure at 700 °C, but they remained orthogonally oriented to each other. No changes occurred in the morphology and growth directions of the Sb:SnO_2_ NWs on r- and a-Al_2_O_3_ by varying the temperature between 700 °C and 1000 °C. In contrast, the epitaxial growth and ordering of the Sb:SnO_2_ NWs was critically dependent on the mass ratio of Sn/Sb. We obtained ordered Sb:SnO_2_ NWs on m-, r- and a-Al_2_O_3_ only for Sn/Sb = 0.15 ± 0.05. One dimensional growth was completely suppressed when Sn/Sb < 0.1, and the Sb:SnO_2_ NWs were not oriented in any particular direction for Sn/Sb > 0.2. Before elaborating further, it is important to mention that we did not obtain any Sb:SnO_2_ NWs at all without adding O_2_ to the flow of Ar during the growth step. In other words, the residual or background O_2_ after purging and the temperature ramp was not significant, and the growth of the Sb:SnO_2_ NWs occurred solely due to the oxygen supplied during the growth step.

Consequently the suppression of one dimensional growth for Sn/Sb < 0.1 is attributed to the total depletion and transfer of Sn into the gas stream during the temperature ramp, before the onset of growth, as it forms a liquid alloy with Sb. Note also that we did not obtain any SbO_2_, Sb_2_O_3_ or Sb_2_O_5_ NWs by using only Sb.

On the other hand the Sb:SnO_2_ NWs were not oriented in any particular direction for Sn/Sb > 0.2. From the above it is clear that an excess of Sb mixed with Sn provides flexible control over the Sn supplied to the Au particles which must be carefully tuned to a minimum for epitaxial growth and ordering to occur. This is further corroborated by the fact that we did not obtain ordered Sb:SnO_2_ NWs without mixing the Sb and Sn even when Sn/Sb ≈ 0.15 ± 0.05.

In addition to the above it is important to point out that the epitaxial growth and ordering of the Sb:SnO_2_ NWs also depends critically on the crystal quality of the m, r and a-Al_2_O_3_ surfaces. It has been shown that m-Al_2_O_3_ is thermodynamically unstable during high-temperature growth and nanostructured grooves composed of s- and r-facets develop along the [112̄0] direction.^35^ In contrast Wang *et al.*^31^ claimed that both the a- and r-plane of Al_2_O_3_ retain their surface structure at elevated temperatures. We annealed the m-, r- and a-Al_2_O_3_ at 1000 °C for 30 min under Ar and O_2_ without any Sn, Sb or Au and observed a drastic reduction in the strength of the XRD peaks of the m- and a-Al_2_O_3_ but the r-Al_2_O_3_ appeared to maintain its surface crystallinity. Subsequently we deposited 1 nm of Au over the pre-annealed m-, r- and a-Al_2_O_3_ and tried to grow epitaxial, ordered Sb:SnO_2_ NWs at 800 °C and 1 mbar using Sn/Sb = 0.1. As expected, we obtained ordered Sb:SnO_2_ only on r-Al_2_O_3_. It appears then, that the deposition of 1 nm Au over pristine m-, r-, and a-Al_2_O_3_ at room temperature, prevents in some way the deterioration of the surface crystal structure and allows the epitaxial growth of ordered Sb:SnO_2_ NWs at elevated temperatures between 700 °C to 1000 °C. In fact, the deposition of a 1 nm Au layer on the m-, r-, and a-Al_2_O_3_, which contain grooves or steps along specific crystallographic directions, leads to instabilities and ruptures of the Au at elevated temperatures as described by Hughes *et al.*^36^ These ruptures occur at high curvature sites, *i.e.*, peaks and ridges, which act as retracting edges leading to a net flux of atoms away from the high positive curvature regions. For sufficiently thin layers, this process exposes the texture or steps of the underlying substrate, and a self-assembly of the Au particles will occur along specific crystallographic orientations.^37^ This in turn will instigate one dimensional epitaxial growth along specific lateral crystallographic directions *via* the VLS mechanism, as shown in Fig. 1(b). When Sn is added to the Au particles, it is expected to reduce the surface tension and contact angle *θ* with the underlying m-, r- and a-Al_2_O_3_ surface, as a consequence of the fact that Sn and Sb have surface tensions of ≈500 mN m^−1^ and 350 mN m^−1^ respectively, but the surface tension of Au is ≈1000 mN m^−1^. A large contact angle implies that the contact area is small, and *vice versa*, a smaller contact angle implies a larger contact area. Consequently, an excess of Sn is expected to lead to the formation of Au–Sn particles having a small contact angle and larger contact area with the underlying m, r and a-Al_2_O_3_ surface, in which case they might not be able to follow the variations in the surface topography which is necessary to obtain ordered Sb:SnO_2_ NWs. The formation of ordered Sb:SnO_2_ NWs is possible due to a reduction of the Sn by the excess of Sb during the temperature ramp, which in turn results into sufficiently small Au–Sn liquid particles that are able to follow steps or grooves on the surface during the growth step at elevated temperatures. Epitaxial growth, then, commences laterally, after which a transition to inclined growth occurs leading to the formation of the ordered networks of Sb:SnO_2_ NWs shown in Fig. 2, 3 and 4.

All of the Sb:SnO_2_ NWs on m-, r- and a-Al_2_O_3_ exhibited clear and well resolved peaks in the XRD, as shown in Fig. 2(d), 3(d) and 4(d) respectively, corresponding to the tetragonal rutile crystal structure of SnO_2_. For comparison, we have included the XRD of the m-, r- and a-Al_2_O_3_ without the Sb:SnO_2_ NWs. More specifically, the Sb:SnO_2_ NWs on m-, r- and a-Al_2_O_3_ exhibit only one or two major peaks in their XRD, consistent with the fact that they grow along specific crystallographic directions. In contrast, we observed a multitude of major peaks from the Sb:SnO_2_ NWs on c-Al_2_O_3_, due to the fact that they do not grow in an epitaxial fashion along specific directions, see ESI.† We did not observe any peaks related to oxides of Sb such as Sb_2_O_3_ or Sb_2_O_5_ which have melting points of 656 °C and 380 °C respectively. In addition, we do not observe any peaks suggesting the formation of Sb_2_O_4_, *i.e.* SbO_2_ which is known to break down into Sb and O_2_ at a higher temperature of 930 °C. Nevertheless the Sb:SnO_2_ NWs contain Sb donor impurities as shown by the EDX spectrum in Fig. 2(e) and as confirmed previously by Raman spectroscopy.^34^ Small differences in the amount of metallic Sb, *i.e.* for Sn/Sb = 0.15 ± 0.05, did not change the crystal structure or orientation of the Sb:SnO_2_ NWs. We obtained exactly the same XRD spectra shown in Fig. 2, 3 and 4 for Sn/Sb = 0.1 and Sn/Sb = 0.15. However, it is important to point out that we did not detect any Sb in the Au particles on the ends of the Sb:SnO_2_ NWs, as shown by the EDX spectrum in Fig. 3(e). This is consistent with the fact that we did not find Sb in the Au after trying to grow Sb_2_O_3_, Sb_2_O_5_ or SbO_2_ NWs, using just Sb, and leads us to suggest that the Sb donor impurities are incorporated into the SnO_2_ NWs by surface diffusion, from their sides, as depicted in Fig. 1(c).

**Fig. 5:**
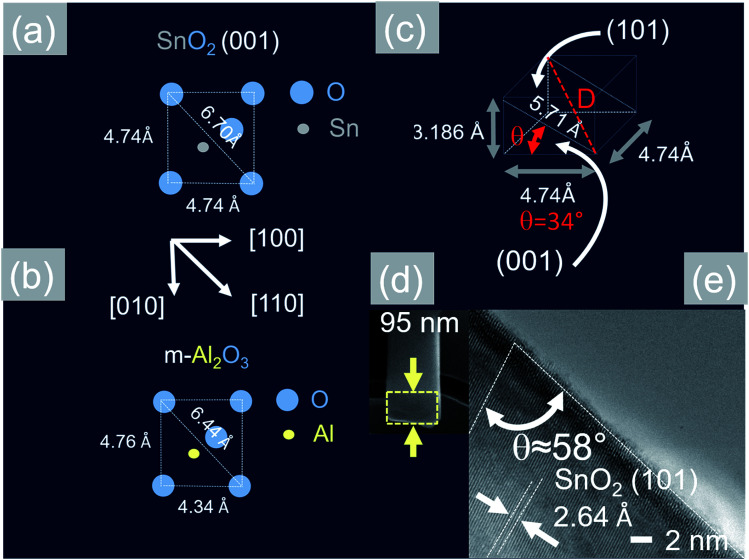
(a) Two dimensional lattice of the SnO_2_ (001) that is grown in an epitaxial way on (b) oxygen terminated m-Al_2_O_3_ (c) crystal structure of tetragonal rutile SnO_2_ (d) TEM and (e) HRTEM images of a Sb:SnO_2_ NW after removing it from the m-Al_2_O_3_.

Now, the Sb:SnO_2_ NWs on m-Al_2_O_3_ exhibited one dominant peak in the XRD, as shown in Fig. 2(d), corresponding to the (002), *i.e.* a multiple of (001), crystallographic planes of tetragonal rutile SnO_2_. The two dimensional lattice of (001) SnO_2_ and the oxygen terminated surface of m-Al_2_O_3_ are shown in Fig. 5(a) and (b) respectively. These have a small lattice mismatch of 0.5% so the Sb:SnO_2_ NWs grow by a stacking of (001) planes on m-Al_2_O_3_ and the in-plane epitaxial relationship is SnO_2_ (001)‖m-Al_2_O_3_. The growth of the Sb:SnO_2_ NWs on m-Al_2_O_3_ is identical to that of SnO_2_ NWs obtained by Leonardy *et al.*^27^ on m-Al_2_O_3_ using SnO as opposed to Sn. For completeness, the tetragonal unit cell of SnO_2_ is shown in Fig. 5(c), from which one may observe that the (101) crystallographic plane of SnO_2_ is inclined at *θ* = 34° with respect to the (001). The Sb:SnO_2_ NWs have rectangular sections, as shown in Fig. 5(d), and grow along the [101] crystallographic direction as confirmed by the HRTEM image of Fig. 5(e). Hence the Sb:SnO_2_ NWs are inclined at *θ* = 34° with respect to the surface of m-Al_2_O_3_, and the fourth fold symmetry of the m-Al_2_O_3_ surface lattice gives rise to Sb:SnO_2_ NWs oriented along two mutually orthogonal directions and the formation of the mesh structure observed in Fig. 2(a)–(c). This has also been confirmed by Wang *et al.*^31^ who showed that un-doped SnO_2_ NWs also grow laterally along two perpendicular directions and cross each other on m-Al_2_O_3_.

**Fig. 6:**
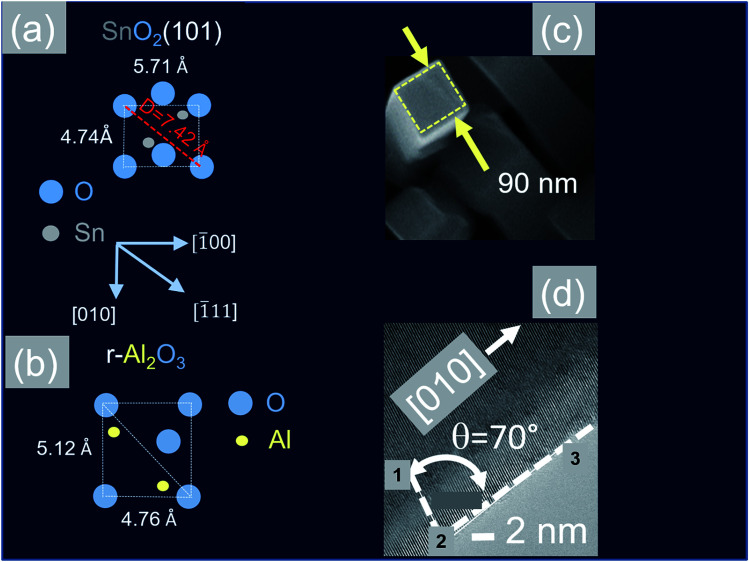
(a) Two dimensional surface lattice of SnO_2_ (101) which grows on (b) oxygen terminated surface of r-Al_2_O_3_ (c) TEM image of Sb:SnO_2_ NWs after removing from r-Al_2_O_3_ (d) HRTEM image of the Sb:SnO_2_ NWs showing the (010) lattice planes.

In contrast to the above we find that the Sb:SnO_2_ NWs on r-Al_2_O_3_ exhibited two dominant peaks in the XRD, as shown in Fig. 3(d), corresponding to the (101) and (202) crystallographic planes of tetragonal rutile SnO_2_. The two dimensional lattice of (101) SnO_2_ and the oxygen terminated surface of r-Al_2_O_3_ are shown in Fig. 6(a) and (b) respectively. These have a larger lattice mismatch of 11%, and the in-plane epitaxial relationship is SnO_2_ (101)‖r-Al_2_O_3_. The growth of the Sb:SnO_2_ NWs on r-Al_2_O_3_ is very similar to the un-doped SnO_2_ NWs of Kim *et al.*^38^ which were also inclined at *θ* = 68° on r-Al_2_O_3_. Lateral SnO_2_ NWs have also been obtained *via* the VLS mechanism by Kim *et al.*^29^ on r-Al_2_O_3_ using C and SnO_2_ as opposed to Sn but they did not observe the transition of growth from lateral to vertical SnO_2_ NWs.

### 3.2 Selective area location growth of Sb:SnO_2_ NWs on r-Al_2_O_3_

**Fig. 7:**
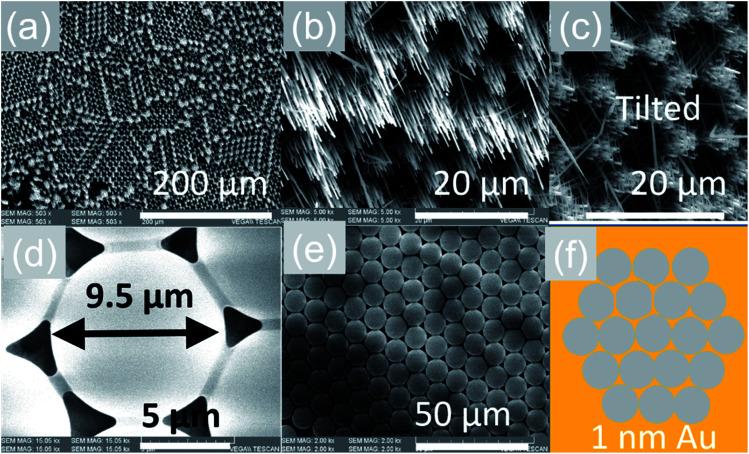
Selective area location growth of Sb:SnO_2_ NWs on r-Al_2_O_3_ (a) low magnification and (b) high magnification image (c) tilted view of the Sb:SnO_2_ NWs on r-Al_2_O_3_ showing that they do not consist two segments as shown in Fig. 3(f); (d) an individual sphere in contact with its six closest neighbors (e) domain of ordered spheres (f) schematic representation of the spheres on r-Al_2_O_3_ after the deposition of 1 nm Au in between the spheres.

The VLS growth mechanism permits selective area location growth on m-, r- and a-Al_2_O_3_ as we did not obtain any Sb:SnO_2_ NWs without using Au. We obtained hexagonally ordered Sb:SnO_2_ NWs on r-Al_2_O_3_ by drop casting ∼10 μl of 9 μm diameter polystyrene spheres on r-Al_2_O_3_, followed by the deposition of a thin layer of ∼1 nm Au as shown in Fig. 7. Subsequently, the spheres were removed in isopropanol by ultrasonic vibration for 1 min, and the Sb:SnO_2_ NWs were grown on the patterned Au on r-Al_2_O_3_ at 800 °C. The Sb:SnO_2_ NWs on r-Al_2_O_3_ do not consist of two segments due to the reduced growth time. One may clearly observe that the Sb:SnO_2_ NWs grow on the r-Al_2_O_3_ in a hexagonal pattern suggesting that one may also obtain different geometries in order to tailor the absorption–transmission spectrum in novel devices such as NWSCs.

### 3.3 Electrical properties of Sb:SnO_2_ NWs on m-, r- and a-Al_2_O_3_

The carrier density of the Sb:SnO_2_ NWs grown on m-, r- and a-Al_2_O_3_ at 800 °C and 1 mbar with Sn/Sb = 0.1 was measured by the Hall effect. We obtained a carrier density of 8 × 10^19^ cm^−3^ which is significantly larger than that measured previously in un-doped SnO_2_ NWs, that was of the order of 10^16^ cm^−3^.^33^ The conductivity of the Sb:SnO_2_ NWs was found to be ≈3 × 10^2^ (Ω cm)^−1^ giving a mobility of 20 cm^2^ V^−1^ s^−1^ which is lower compared to 70 cm^2^ V^−1^ s^−1^ in un-doped SnO_2_ NWs that was previously measured by THz conductivity spectroscopy.^33^ We did not observe a significant variation in the carrier density of the Sb:SnO_2_ NWs on m-, r- and a-Al_2_O_3_, or due to slight variations of Sn/Sb = 0.15 ± 0.05. We measured the Hall effect of the Sb:SnO_2_ NWs obtained with Sn/Sb = 0.15 and found a carrier density of 8.3 × 10^19^ cm^−3^ which is very close to that obtained with Sn/Sb = 0.1. However the carrier density in the Sb:SnO_2_ NWs obtained at 900 °C was smaller *i.e.* 6 × 10^18^ cm^−3^ while those obtained at 1000 °C had an even smaller carrier density of 4 × 10^17^ cm^−3^ and conductivity of ≈3 (Ω cm)^−1^. This is attributed to the transfer of the Sb into the gas stream during the temperature ramp, and before the onset of one dimensional growth, due to the high vapor pressure of Sb, which in turn limits the supply and incorporation of Sb impurities into the SnO_2_ NWs during the growth step. This trend is consistent with the findings of Klamchuen *et al.*,^22^ who found that the doping level in SnO_2_ NWs grown at 650 °C was twice that obtained at 750 °C, attributed to a suppression of impurity re-evaporation. They also observed that the doping level did not increase further upon reducing the growth temperature from 650 °C to 550 °C, which was explained by a suppression of the diffusion length of impurity ad-atoms on the surface of the SnO_2_ NWs, and the presence of a temperature activated energy barrier which is necessary for the incorporation of the Sb impurities into the host lattice of SnO_2_.

We suggest that the incorporation of Sb impurities into the SnO_2_ NWs occurs through the sides of the SnO_2_ NWs by thermal diffusion, or, *via* the triple phase junction, as shown in Fig. 1(c), similar to the mechanism proposed by Klamchuen *et al.*^22^ This is also corroborated by the fact that we did not detect any Sb in the Au particles after attempting to grow SbO_2_, Sb_2_O_3_ or Sb_2_O_5_ NWs on r-Al_2_O_3_, by using only Sb, and also by the fact that we did not detect any Sb in the Au:Sn particles on the ends of the Sb:SnO_2_ NWs, as shown in Fig. 3(e). It is also consistent with the findings of McGinley *et al.*,^39^ who showed that the surface of SnO_2_ nanoparticles is terminated by an oxygen rich layer, but when doped n-type with 9% or 17% Sb, the impurity atoms are concentrated near the surface of the SnO_2_ nanoparticles with an oxidation state of five.

**Fig. 8:**
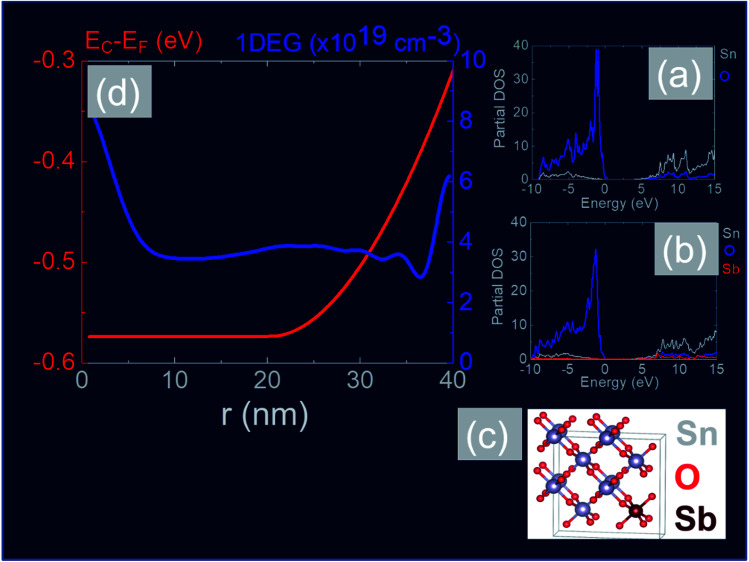
(a) DOS for un-doped SnO_2_ and (b) DOS for Sb:SnO_2_ with 12.5 at% (≡4 × 10^21^ cm^−3^) (c) structural model showing Sb substitutional donor impurity in SnO_2_ (d) SCPS CB potential profile (red) and 1DEG charge distribution (blue) *versus* distance along the radial direction of a Sb:SnO_2_ NWs with a diameter of 80 nm taking *E*_C_ − *E*_F_ = −0.3 eV at the surface and assuming a uniform distribution of ionized donor impurities *N*_D_^+^ = 1 × 10^19^ cm^−3^ between *r* = 20 and 40 nm.

In order to obtain a more detailed understanding of the electronic properties of the Sb:SnO_2_ NWs we carried out electronic structure calculations from first principles using the CASTEP plain wave DFT code^40,41^ and the Heyd–Scuseria–Ernzerhof (HSE)^42^ exchange correlate-correlation energy functional. After relaxation of the cell, we obtained lattice constants of *a* = *b* = 4.82 Å and *c* = 3.23 Å which are in close agreement to reported values.^42^ DOS calculations were performed for the case of (a) the perfect SnO_2_ cell and (b) the Sb-doped SnO_2_ cell, shown in Fig. 8(a) and (b) respectively. The maximum of the valence band (VB) is set at zero energy level. It is evident from the partial density of states shown in Fig. 8(a) and (b) that the VB is dominated by O and the CB by Sn.^60^ The band gap was found to be 3.6 eV for the perfect structure but a slight reduction of the band gap down to 3.5 eV was observed with doping. This semi-empirical method yields a much more accurate band gap than calculations performed based on the Perdew–Burke–Ernzerhof (PBE) GGA functional which are not shown here. In addition we find that the incorporation of Sb into the SnO_2_ crystal does not produce any deep levels within the band gap which are in general detrimental to the operation of optoelectronic devices such as solar cells, light emitting diodes *etc.* In other words the Sb impurities are incorporated into the SnO_2_ lattice as substitutional donors and our calculations show that the Fermi level resides 0.46 eV above the CB edge for 12.5 at% Sb (≡4 × 10^21^ cm^−3^).

In addition, we calculated the conduction band (CB) potential profile, and one dimensional electron gas (1DEG) charge distribution, along the radial direction, *via* the self-consistent solution of the Poisson–Schrödinger (SCPS) equations, in the effective mass approximation, as described in detail elsewhere.^43^ The SCPS calculations were carried out by taking into account the effective mass and dielectric constant of SnO_2_ , *i.e.*
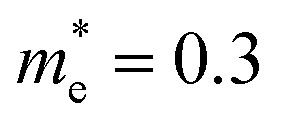
 (ref. 44 and 45) and *ε*_r_ = 13.5 (ref. 46 and 47) respectively. In order to obtain a carrier density of 8 × 10^19^ cm^−3^ as determined from the Hall effect we have taken the Fermi level to be energetically located ≈0.3 eV above the CB edge, at the surface of the Sb:SnO_2_ NWs. This is consistent with the electronic structure calculations of Mishra *et al.*,^48^ who showed that the Sb impurities in SnO_2_ form a band, which has an energetic overlap with the conduction band and a nearly free electron structure, *i.e.* behaves like a metallic band. It is also consistent with Li *et al.*,^49^ who calculated the electronic structure of Sb:SnO_2_ for 6.25% Sb, and found that the Fermi level moves into the CB upon the incorporation of Sb, and displays metallic character, but also with Farahani *et al.*,^50^ who showed that the Fermi level resides ≈0.33 eV above the CB edge at the surface of Sb:SnO_2_ epitaxial layers grown by molecular beam epitaxy on r-Al_2_O_3_.

**Fig. 9:**
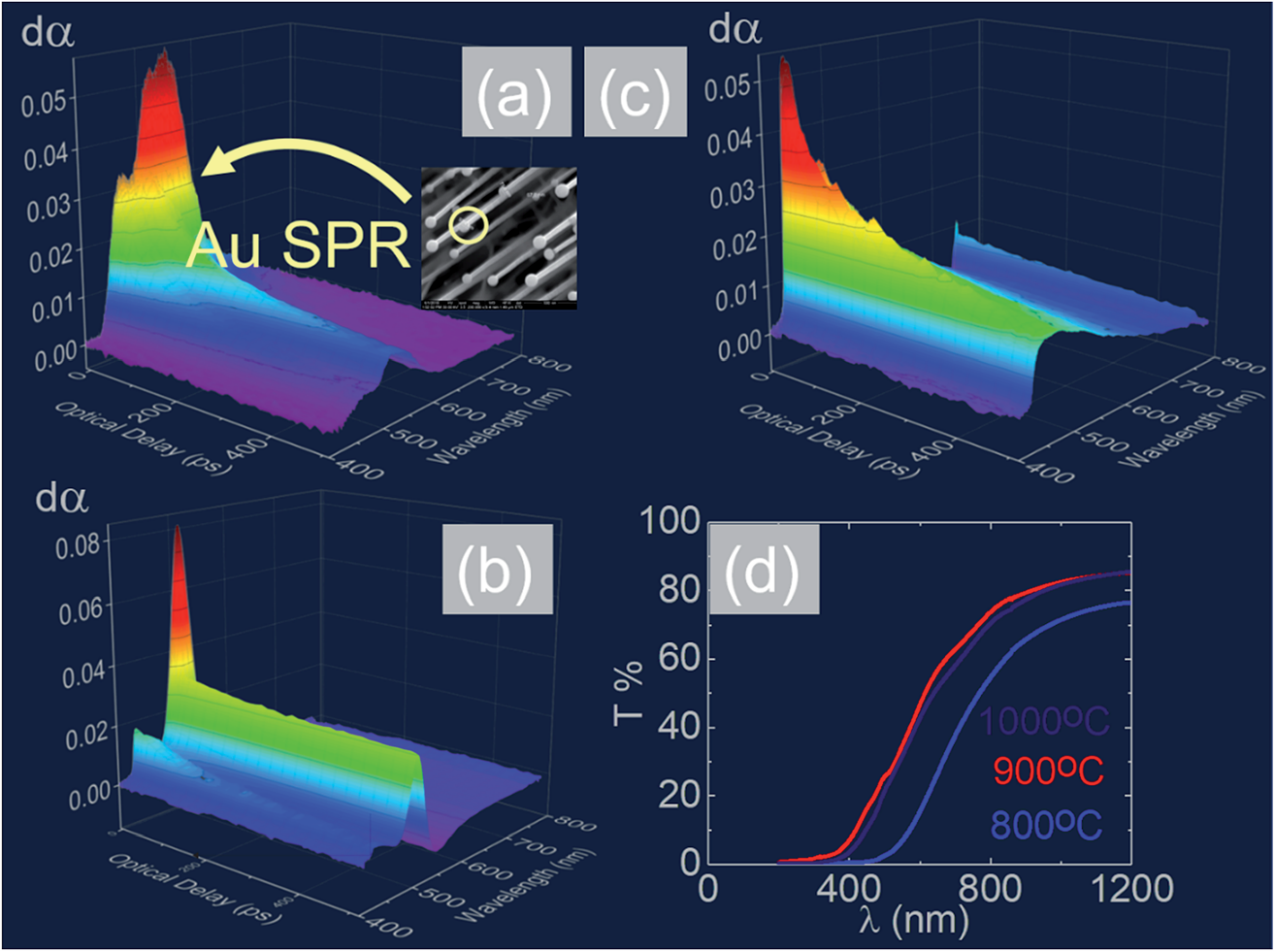
Ultrafast, differential transient absorption d*α versus* optical delay *τ* (ps) and probe wavelength *λ* (nm) through the Sb:SnO_2_ NWs grown on r-Al_2_O_3_ at (a) 800 °C, (b) 900 °C and (c) 1000 °C; (d) steady state transmission of light through the Sb:SnO_2_ NWs grown on r-Al_2_O_3_ at different temperatures.

The CB potential profile with respect to the Fermi level, *i.e. E*_C_ − *E*_F_ and 1DEG charge distribution, *versus* distance along the radial direction of the Sb:SnO_2_ NWs, is shown in Fig. 8(d), where we have taken the donor impurities to be confined between *r* = 20 and 40 nm. The CB edge potential profile is near flat band in the vicinity of the core and the 1DEG charge distribution has a maximum at the core and a local maximum at the surface. It should be noted that we obtain similar band profiles and charge distributions by taking a uniform distribution of Sb impurities throughout the Sb:SnO_2_ NWs. However the 1DEG in the vicinity of the core is expected to have a higher mobility when the density of donor impurities is larger at the surface than the core, as is the case in Fig. 9(d). In other words, the incorporation of Sb impurities into the SnO_2_ NWs *via* surface diffusion is not a drawback in the end. We estimate that the mobility in the Sb:SnO_2_ NWs with a maximum carrier density of the order of 10^20^ cm^−3^ is a few tens of cm^2^ V^−1^ s^−1^ (ref. 23 and 24) so the resistivity of the Sb:SnO_2_ NWs is of the order of 10^−3^ Ω cm which in turn is attractive for the deposition of metal contacts with low resistance and the fabrication of high performance devices.

### 3.4 Optical properties of Sb:SnO_2_ NWs on m-, r- and a-Al_2_O_3_

In the past the transmission of light through Sb:SnO_2_ NWs^14–24^ has not been investigated as they were not ordered or oriented along any particular direction which in turn leads to a considerable suppression of transmission and transparency. In contrast a higher transmission of light is expected to occur through ordered MO NWs. We measured the steady state transmission through the Sb:SnO_2_ NWs that were grown at 800 °C on r-Al_2_O_3_ as shown in Fig. 9(d). The Sb:SnO_2_ NWs have an optical transmission of 80% above 800 nm but they absorb light in the visible between 400 nm to 800 nm. We do not observe any interference effects since the spacing between the Sb:SnO_2_ NWs is considerably smaller than the wavelength of light. In addition we find that the Sb:SnO_2_ NWs on r-Al_2_O_3_ obtained at 900 °C and 1000 °C have a higher transparency compared to those obtained at 800 °C due to their shorter lengths. In order to understand the origin of the absorption we have measured the transient absorption through the Sb:SnO_2_ NWs on an fs time scale as shown in Fig. 9(a)–(c). In all cases, we observe a strong peak in the time evolution of the differential absorption between 500 nm and 600 nm attributed to the surface plasmon resonance (SPR) of the Au–Sn particles, corresponding to sizes between 10 nm and 100 nm (ref. 51) and the existence of surface states lying energetically in the energy gap of SnO_2_.^52^ One may observe a slight blue shift of the peak from 900 °C to 1000 °C in Fig. 9(b) and (c), consistent with that observed in the steady state transmission, that might be related to the smaller size of the Au–Sn particles due to the depletion of Sn that occurs during the temperature ramp and/or the elimination of mid gap states at elevated temperatures. It is important to point out, that the transparency of the Sb:SnO_2_ NWs on r-Al_2_O_3_ may be increased further by selective area location growth as shown in Fig. 7 which leads to a higher transmission through the voids between the Sb:SnO_2_ NW.

**Fig. 10:**
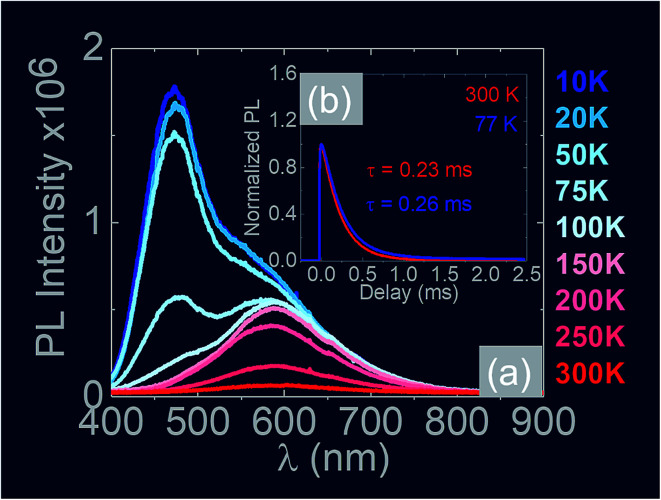
(a) Temperature dependence of the PL obtained from the Sb:SnO_2_ NWs grown on r-Al_2_O_3_ at 800 °C (b) time resolved PL taken at *λ* = 600 nm for 300 K and 77 K along with the corresponding lifetimes.

Finally it is worthwhile considering the PL obtained from the Sb:SnO_2_ NWs, grown on r-Al_2_O_3_ at 800 °C, as shown in Fig. 10(a), in which case we observe emission at *λ* = 600 nm (≡2.1 eV) and 300 K. Bulk SnO_2_ has a direct energy band gap of 3.7 eV but the even – parity symmetry of the conduction-band minimum and valence-band maximum states prohibits band – edge radiative transitions which has limited the use of SnO_2_ for the fabrication of light emitting diodes. The PL at 2.1 eV is attributed to radiative recombination between deep donor and acceptor like states residing energetically in the energy band gap of SnO_2_ that are related to oxygen vacancies. We observe a suppression of the maximum at *λ* = 600 nm and the emergence of emission at *λ* = 470 nm (≡2.6 eV) by decreasing the temperature from 300 K to 10 K due to radiative recombination *via* shallower levels as proposed by Luo *et al.*^53^ However an interesting aspect of the PL emission at 2.1 eV and 2.6 eV is that it has a lifetime of *τ* ≈ 0.2 ms as shown by the time resolved PL in Fig. 10(b). This is considerably higher than the lifetimes extracted from SnO_2_ rods and particles which are of the order of 100 ns (ref. 54) and comparable to Eu doped SnO_2_ nanocrystals.^55^ Hence, in principle, the Sb:SnO_2_ NWs described here may be processed into devices capable of light emission but also NWSCs.^56,57^

To the best of our knowledge no one has previously used ordered networks of Sn:In_2_O_3_ or Sb:SnO_2_ NWs to make NWSCs despite the fact that Battaglia *et al.*^58^ has showed that periodic photonic nanostructures outperform their random counterparts in trapping light in solar cells. It is desirable then to use these highly conductive, ordered networks, of Sb:SnO_2_ NWs in order to improve the performance of all-solid state NWSCs. In addition the Sb:SnO_2_ NWs can be used in perovskite solar cells as an electron transport layer (ETL). According to Jiang *et al.*^59^ a traditional ETL such as TiO_2_, is not very efficient for charge extraction at the interface, especially in planar structures. In addition, the devices using TiO_2_ suffer from serious degradation under ultraviolet illumination. SnO_2_ shows a better band alignment with the perovskite absorption layer and higher electron mobility, which is helpful for electron extraction. Consequently the specific ordered networks of Sb:SnO_2_ NWs described here may serve as a scaffold on top of which one may deposit a perovskite absorber layer and a hole transport layer in order to create a p–n junction solar cell.

## 4 Conclusions

We have grown epitaxial, ordered Sb:SnO_2_ NWs *via* the VLS mechanism on m-, r- and a-Al_2_O_3_ between 700 °C and 1000 °C using metallic Sn containing an excess of Sb *i.e.* for Sn/Sb = 0.15 ± 0.05 under a flow of Ar and O_2_ at 1.5 ± 0.5 mbar. One dimensional growth was suppressed for Sn/Sb < 0.1 while the Sb:SnO_2_ NWs were not oriented along any particular direction for Sn/Sb > 0.2. Consequently highly conductive and directional Sb:SnO_2_ NWs may only be obtained in a narrow window of growth conditions. All of the Sb:SnO_2_ NWs have the tetragonal rutile crystal structure and square sections. The Sb:SnO_2_ NWs are oriented along two mutually perpendicular directions forming a rectangular mesh on m-Al_2_O_3_ with a maximum lattice mismatch of 0.1%. In contrast the Sb:SnO_2_ NWs on r-Al_2_O_3_ are all oriented in one direction but have a larger lattice mismatch of 10%. The morphology and growth directions of the Sb:SnO_2_ NWs on m-, r- and a-Al_2_O_3_ did not change by varying the growth temperature between 700 °C and 1000 °C but the carrier density changed from 8 × 10^19^ cm^−3^ to 4 × 10^17^ cm^−3^ due to the re-evaporation and limited incorporation of Sb donor impurities into the SnO_2_ NWs with increasing temperature. All of the Sb:SnO_2_ NWs had a high transmission of 80% above 800 nm and absorbed light between 400 nm to 800 nm primarily due to the SPR of the Au particles. The transmission may be improved significantly by selective area location growth which we have shown that is possible on r-Al_2_O_3_ by patterning the catalyst. In addition the Sb:SnO_2_ NWs on m-, r- and a-Al_2_O_3_ are capable of light emission with remarkably long lifetimes of 0.2 ms and are attractive for the realization of NWSCs.

## Conflicts of interest

There are no conflicts of interest to declare.

## Supplementary Material

NA-001-C9NA00074G-s001
