# Supplementary material for: Epitaxial highly ordered Sb:SnO2 nanowires grown by the vapor liquid solid mechanism on m-, r- and a-Al2O3
Source: Nanoscale Adv. 2019 Apr 9;1(5):1980–90. doi: 10.1039/c9na00074g (PMC9419487; doi:10.1039/c9na00074g)
Supplement: NA-001-C9NA00074G-s001 [file NA-001-C9NA00074G-s001.pdf]

Electronic Supplementary Material (ESI) for Nanoscale Advances.
This journal is © The Royal Society of Chemistry 2019

Supporting Information

Epitaxial Highly Ordered Sb: SnO2 Grown by the Vapor Liquid Solid Mechanism on m-, r- and a-Al2O3

M. Zervos □[1] , N. Lathiotakis ᴷ, N. Kelaidis ˡ, A. Othonos §, E. Tanasă ǁ, E. Vasile ǁ

□ Nanostructured Materials and Devices Laboratory, School *Of* Engineering, University *of* Cyprus, PO Box 20537, Nicosia, 1678, Cyprus

ᴷ Theoretical and Physical Chemistry Institute, National Hellenic Research Foundation, Vass. Constantinou 48, GR-11635 Athens, Greece.

ˡ Faculty of Engineering, Environment and Computing, Coventry University, Priory Street, Coventry CV1 5FB, United Kingdom

§ Laboratory *Of* Ultrafast Science, Department *Of* Physics, University *of* Cyprus, P.O.Box 20537, Nicosia, 1678, Cyprus.

ǁ Department of Science and Engineering of Oxide Materials and Nanomaterials, Politehnica University of Bucharest, 313 Splaiul Independentei, Bucharest, 060042, Romania

[1] E-mail:zervos@ucy.ac.cy

S1 Properties of Sb: SnO_2 NWs obtained on c-Al_2O_3

The Sb: SnO_2 NWs that were grown on c-Al_2O_3 at 800°C using Sn/Sb = 0.15 ± 0.05 under a flow of Ar and O_2 at 1 ± 0.5 mbar were not ordered as shown in Figure S1 (a). This is in contrast to the findings of Mazeina *et al.* [1] who obtained vertical SnO_2 NWs via the VLS mechanism on c-Al_2O_3 at 900°C albeit with limited ordering and uniformity. Nevertheless we observed the formation of ordered Sb: SnO_2 NWs on the sides of the c-Al_2O_3 as shown in Figure S1 (b). In order to find out if this was related to differences in the thickness of the Au on the top and sides of the c-Al_2O_3 we deposited 1 nm of Au directly on the side of the c-Al_2O_3 as shown in Figure S1(c) and repeated the growth keeping all else equal. However we did not obtain oriented Sb: SnO_2 NWs on top of c-Al_2O_3, only on its side. It should also be mentioned that we did not observe any ordering of the Sb: SnO_2 NWs on c-Al_2O_3 by changing its inclination or angle with respect to the gas flow. This is consistent with the findings of Kim *et al.* [2] who investigated the lateral epitaxial growth of faceted and aligned SnO_2 NWs on r-Al_2O_3 and found little or no dependence of the alignment on the gas flow direction. The XRD spectrum of the Sb: SnO_2 NWs on c-Al_2O_3 is shown in Figure S1 (e) from which we observe the occurrence of many peaks belonging to the tetragonal rutile crystal structure of SnO_2. The Sb: SnO_2 NWs grow in many different crystallographic directions as they do not obey a strict epitaxial relation with the underlying c-Al_2O_3 but nevertheless are highly crystalline. In contrast the XRD spectrum of the ordered Sb: SnO_2 NWs obtained on the sides is shown in Figure S1 (d) from which we observe just two peaks corresponding to the tetragonal rutile crystal structure of SnO_2 and a-Al_2O_3. This is due to the fact that the Sb: SnO_2 NWs grow in

an epitaxial fashion on the side facet along a specific crystallographic direction. The ordered Sb: SnO_2 NWs on the side of c-Al_2O_3 are also highly crystalline but it is not possible to use them for the realization of devices such as nanowires solar cells so we did not investigate further their structural properties. This prompted us to grow Sb: SnO_2 NWs on top of m-, r- and a-Al_2O_3 in which case the ordered Sb: SnO_2 NWs can be processed further into devices.

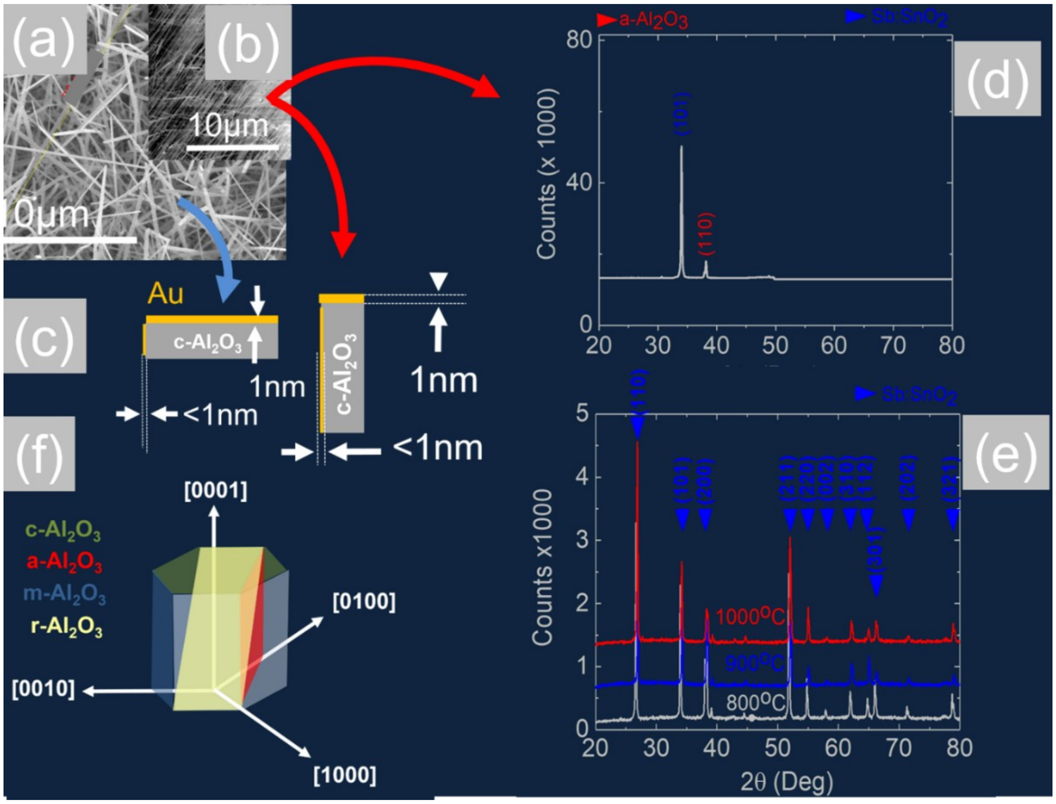

Figure S1. (a) SEM images of the Sb: SnO_2 NWs obtained at 800°C on top of c-Al_2O_3 and (b) on the side of c-Al_2O_3, with Sb/Sn = 0.1 (c) schematic of the deposition of Au on top of c-Al_2O_3 and on its side. The corresponding XRD of (a) and (b) are shown in (d) and (e) respectively. The Sb: SnO_2 NWs grow in an ordered fashion on the vertical a-Al_2O_3 side facet which is depicted schematically in relation to c-Al_2O_3 in (f).

References

(1) Mazeina, L.; Picard, Y.N.; Caldwell, J.D.; Glaser, E.R.; Prokes, S.M. Growth and Photoluminescence Properties of Vertically Aligned SnO_2 Nanowires. *J. Cryst. Growth* **2009**, 311 3158–3162

(2) Kim, W.S.; Kim, D.; Hong, S.H. Lateral Epitaxial Growth of Faceted SnO_2 Nanowires with Self Alignment. *Cryst. Eng. Comm.* **2014,** 16, 9340-9344
